# Genomic and Functional Analysis of Two Halophilic IAA-Producing *Vreelandella* Strains

**DOI:** 10.1007/s00284-026-04946-7

**Published:** 2026-05-19

**Authors:** Gianmaria Oliva, Bruno Hay Mele, Concetta Di Lorenzo, Mimmo Turano, Stefano Castiglione, Giovanni Vigliotta

**Affiliations:** 1 Department of Chemistry and Biology “A. Zambelli”, University of Salerno, Fisciano, 84084 SA Italy; 2 Department for the Promotion of Human Science and Quality of Life, San Raffaele Open University, Rome, 00166 RM Italy; 3 Department of Biology, University of Naples Federico II, Naples, 80126 NA Italy

## Abstract

Soil salinization poses a crucial challenge to agriculture; in fact, it reduces both growth and crop yields. *Vreelandella* species emerged as promising plant-growth-promoting-rhizobacteria (PGPR) capable of thriving in saline environments. Our study illustrates a comparative genomic analysis of two *Vreelandella* species: *Vreelandella titanicae* (QH24) and *Vreelandella alkaliphila* (QH23), isolated from the rhizosphere of quinoa cultivated in the presence of salt. Genome sequencing, functional annotation, and protein interaction network modeling were used to explore their adaptive mechanisms to salinity and their potential to plant growth promotion, with a specific focus on indole-3-acetic acid (IAA) biosynthesis under salt stress. Both genomes revealed several genes involved in osmotic stress response, including those associated with ectoine, glycine betaine, and proline metabolism. PGPR-related genes, particularly those involved in IAA biosynthesis, siderophore production, and phosphate metabolism, were identified in both species, although key differences emerged. In vitro assays on quinoa seedlings confirmed the PGPR potential of both strains, showing improved seedling growth and modification in root architecture. Moreover, both the strains produced more IAA in response to increasing amounts of NaCl, with QH23 exhibiting significant higher quantities respect to QH24. Network analysis revealed that *ald*A acts as a central regulatory hub linking auxin biosynthesis and salt-responsive metabolism in QH24, whereas *ami*E in QH23 is part of a wider interaction module. These findings indicate that QH24 and QH23 take different genetic strategies to coordinate IAA synthesis under saline conditions. Our results provide important information regarding the genomic basis of PGPR activity as a response to saline conditions and lay the foundations for further transcriptomic and metabolomic studies to optimize PGPR use in sustainable agricultural practices.

**Supplementary Information:**

The online version contains supplementary material available at 10.1007/s00284-026-04946-7.

## Key points

- *V. titanicae* and *V. alkaliphila* improve the growth and root architecture of quinoa.
- *V. alkaliphila* shows enhanced salt-induced IAA biosynthesis compared to *V. titanicae*.
- *ald* A regulate the IAA production in *V. titanicae*, while *ami*E in *V. alkaliphila*.

**Supplementary Information:**

The online version contains supplementary material available at 10.1007/s00284-026-04946-7.

## Introduction

Increasing soil salinity represents one of the major threats to global food security, with significant impacts on crop productivity and soil quality. About 20% of the world’s cultivated land and 33% of irrigated land are by now affected by soil salinization, a condition thus limiting nutrient uptake and, consequently, compromising crop yields [1]. This problem is expected to worsen due to climate change, sea level rise and intensive agricultural practices that favor the accumulation of salts in cultivated soils [2]. Salty environments, such as saline soils, salt marshes, coastal lagoons, and deep marine sediments, host a highly specialized microbial community capable of surviving in extreme conditions [3]. Among these microorganisms, bacteria of the *Vreelandella* genus (class γ-proteobacteria, family *Halomonadaceae*), represents a fundamental resource for understanding adaptive mechanisms to salinity and for developing of innovative strategies to manage saline soils and improve agricultural productivity [4, 5].

Halophilia and halotolerance are cellular properties that characterize microorganisms capable of surviving and proliferating in environments where salt (NaCl) concentrations are higher than normal, up to saturation in the case of extremophiles. Halotolerant microorganisms can live at high NaCl concentrations, while the halophilic microorganisms necessarily require its presence. The minimum salt concentration, as well as the optimal or maximum tolerated concentration, depend on the organism and has a very high range of variability, which makes a precise classification based on these parameters almost difficult. Despite this, the various categories are distinguished based on NaCl concentration ranges: slight halophiles show optimum growth at 2–5% NaCl, moderate halophiles at 5–20% NaCl, and extreme halophiles show 20–30% NaCl [6]. *Vreelandella* species are typically moderate halophiles and adopt adaptive strategies that include the accumulation of compatible osmolytes such as glycine, betaine, ectoine and trehalose, as well as the balancing of intracellular ions to maintain cellular stability [7]. This ability to survive in saline conditions makes them suitable for biotechnological applications especially in agriculture in degraded soils such as saline ones.

In this regard, a key role is played by Plant Growth-Promoting Rhizobacteria (PGPR), a group of bacteria that interact with plants promoting their growth and resistance to abiotic stress [8]. PGPR improve the availability of essential nutrients, such as nitrogen and phosphorus, produce phytohormones and protect plants from pathogens and environmental stress [9]. In saline soils, their action is particularly relevant because they promote plant growth by reducing the deleterious effects of salinity through the production of osmoprotectant, the regulation of plant hormones as indole-3-acetic acid (IAA), and the synthesis of 1-aminocyclopropane-1-carboxylate (ACC) deaminase, an enzyme that degrades the precursor of ethylene, the plant stress hormone [10]. Many species belonging to the genus *Vreelandella* have been shown to possess genes related to these plant-growth promoting functions [11]. However, although these bacteria are salt resistant, NaCl can still affect their plant growth-promoting activities, particularly the biosynthesis of IAA. This aspect has been only minimally explored in the scientific literature. In this regard, our preliminary study showed that NaCl differentially regulates IAA production in two strains of the genus *Vreelandella* [12]. Understanding these regulation dynamics is crucial for optimizing the use of *Vreelandella* strains, already available at our department, in agricultural applications.

Among *Vreelandella* species, *Vreelandella titanicae* and *Vreelandella alkaliphila*, once known as *Halomonas titanicae* and *Halomonas alkaliphila*, and recently renamed as *Vreelandella* [13], represent two significant examples of bacterial species well adapted to saline conditions, for convenience we will call the two species all along the text *V. titanicae* and *V. alkaliphila*.

*V. titanicae* was isolated from the wreck of the transatlantic Titanic and known for its ability to degrade ferrous materials in deep saline environments [14, 15]; while, *V. alkaliphila* is known to grow in highly alkaline environments [16].

Comparative genome analysis of the two *Vreelandella* strains can provide insights into the adaptation mechanisms to saline environmental conditions, as well as their potential biotechnological application. To date, many studies have focused on phenotypic and biochemical characterization of these microorganisms, and only a limited part of the known species has had its entire genome sequenced. Furthermore, the genetic mechanisms at the basis of salt homeostasis regulation, the synthesis of secondary metabolites and their interaction with other living organisms remain partly unexplored. Understanding these aspects is essential not only to fill the gaps in the biology of extremophile microorganisms, but also to fully exploit their applicative potential.

In this study, we sequenced the genomes of *V. titanicae* strain QH24 (reported in the text as QH24) and *V. alkaliphila* strain QH23 (reported in the text as QH23) and subsequently analyzed them through functional annotation and genomic comparison. Our aim was to identify the main genetic features that allow these bacteria to survive under high salinity and investigate the genes responsible for key plant growth-promoting activities. We focused our attention particularly on genes involved in IAA biosynthesis and their regulation by NaCl, integrating our genomic findings with experimental data on IAA production under increasing NaCl concentrations. Finally, we tested in vitro these bacterial strains to demonstrate their role in plant growth promotion.

## Materials and Methods

### Isolation and Characterization of Salt-Tolerant Bacteria

QH24 and QH23 were isolated from *Chenopodium quinoa* Willd. (cv. Regalona) rhizosphere at the Plant Biology Laboratory of the University of Salerno (Campania, Italy) in our previous work and characterized morphologically, biochemically and for the main plant growth promoting (PGP) features [12].

### DNA Extraction and Whole Genome Sequencing Analysis

Genomic DNA of each strain was extracted from fresh overnight culture using DNeasy PowerSoil Pro Kit (QIAGEN, Hilder, Germany), according to the manufacturer’s instruction. DNA quality and quantity were assessed electrophoretic and spectrophotometric analysis (by UV absorption analysis at 280, 260 and 230 nm) using the NanoDrop 2000 UV-Vis spectrophotometer (Thermo Scientific, Waltham, USA). High-quality genomic DNA was stored at −20 °C for further DNA analysis. Whole genome sequencing was realized by shotgun procedure by Genomix4Life SrL (Baronissi, Italy).

### Genome Assembly, Gene Prediction, and Functional Annotation

Raw sequencing reads were quality-filtered and trimmed using CLC Genomics Workbench v25.0 software (QIAGEN, Hilder, Germany), with quality checks setting default parameters. Even in CLC software, De novo genome assembly was performed using the “De novo assembly” module with default settings. The assembled genome was processed for gene prediction and annotations using Prokka v1.14.6 software [17] with both default bacterial database and parameters, as well as Cluster of Orthologous Groups (COG), Mobile Genetic Elements (MGEs), probable Antibiotic Resistance Genes (ARGs) and CRISPR regions were analyzed. A circular genome map for each strain was obtained using genome annotation file on Proksee [18] and many features were showed (CDS, tRNA, rRNA, GC content, GC skew+−).

### Phylogenetic Analysis

The sequences of our isolates were identified by a similarity search using the Basic Local Alignment Search Tool platform (BLAST) function of GenBank at the National Center for Biotechnology Information (NCBI; https://www.ncbi.nlm.nih.gov, accessed on 8 June 2025). The phylogenetic analysis was realized using the software MEGA11 [19]. The 16 S rDNA sequences (1300–1500 nucleotides) of 20 type strains were collected from GenBank datasets and aligned by Clustal W software with that of our strains. Phylogenetic analysis and tree construction were performed using the Kimura two-parameter algorithm and the neighbor-joining method. The robustness of the inferred phylogenies was determined by bootstrap analysis based on 1000 data resampling’s. Dichotomies supported by bootstrap scores > 50 were considered significative [20].

### Plant Growth Promoting Assay on Quinoa Seedlings

To demonstrate that QH23 and QH24 strains were PGPR, in vitro experiments were performed on quinoa seeds. Briefly, surface-sterilized quinoa seeds (*Chenopodium quinoa* Willd. cv. Regalona) were sown on Murashige and Skoog (MS) modified agar plates (in gL^− 1^: (NH_4_)NO_3_ 2.0, MgSO_4_⋅7H_2_O 0.2, FeSO_4_⋅7H_2_O 0.0001, CaCl2⋅2H_2_O 0.05, glucose 2.0, Na_2_HPO_4_ 0.6, KH_2_PO_4_ 0.4, tryptophane 0.02, arginine 0.02, agar 8.0) pre-inoculated with QH23 and QH24. The bacterial treatment was carried out by two different approaches: (i) diffusion plating, (ii) inclusion method. In the diffusion assay, bacterial suspension was spread onto the agar surface, while in the inclusion assay, bacterial cultures were mixed into the molten MS medium (at 45 °C). In both cases, after inoculation, the seeds were placed on the agar surface. Each treatment consisted of 5 seeds, and the germination rate was ~ 80% (on average 4 out of 5 seeds per treatment).

After initial preparation, the plates are transferred to a climate room with constant humidity (60%) and temperature (24 °C), setup with a photoperiod of 16 h of light and 8 h of dark. After 10 days of development the seedlings were removed for morphometric characterization by measuring their length and observing the roots under a light microscope. Results were compared with experimental controls prepared without bacterial inoculation.

### Growth Kinetics Studies at Different NaCl Concentration

Bacterial growth kinetics were conducted in Erlenmeyer flask of 250 mL, in triplicate. Briefly, 0.01 OD_600_ (optical density at 600 nm) of overnight bacterial culture was transferred in each flask containing 50 mL of modified Marine-Broth (in g L^− 1^: MgCl_2_ 6H_2_O, 5.9; yeast extract, 0.1; Na_2_SO_4_, 3.24; CaCl_2_ 2H_2_O, 1.8; NaHCO_3_, 0.16; KCl, 0.55; glucose, 1.0; tryptophan, 0.5; FeSO_4_ 6H_2_O, 0.001; KBr, 0.08; H_3_BO_3_, 0.022; Na_2_HPO_4_, 0.008; (NH_4_)NO_3_, 0.0016; SrCl, 0.034,), and three different NaCl concentrations (0, 0.5, 1.0 M). The condition without NaCl (0 M) corresponds to the basal salinity of the modified Marine-Broth medium, as described above.

The supplementation with 0.5 g L^− 1^ tryptophan was selected based on our previous work, in which we demonstrated that this concentration corresponds to the highest IAA production in closely related strains [21]. Although this level exceeds typical environmental concentrations, it ensures activation of the tryptophan-dependent IAA biosynthetic pathways and allows controlled comparative analysis of salt-responsive regulation. Moreover, also the NaCl concentrations and the 48 h time point were selected based on our previous fermentation study, in which a broader kinetic and optimization analysis was performed. In the present work, the experimental design was intended to allow standardized comparison between strains rather than to determine the optimal salt concentration or the full temporal dynamics of IAA biosynthesis.

The flasks were incubated, at 28 °C, with constant shaking at 220 rpm (New Brunswick Innova 43 Incubator Shaker, Eppendorf, Milan, Italy). The growth was measured spectrophotometrically up to 48 h, by determination of optical density at 600 nm. The IAA production was measured through Salkowski method and OD measuring at 530 nm after 48 h of growth [22].

### Analysis of Gene/Protein Interaction Networks

To explore potential functional relationships and compare QH24 and QH23, we built and analyzed gene/protein interactions networks, with a focus on genes involved in of IAA biosynthesis and NaCl adaptation. Based on the availability of *Vreelandella/Halomonas* species on STRING database (Search Tool for the Retrieval of Interacting Genes/Proteins v2.2.0; https://string-db.org), we selected some different closely related species: *Halomonas elongata*, *Vreelandella* sp. KO116, *Vreelandella hydrothermalis*,* and Halomonas* sp. TD01, as reference to generate the interaction networks.

The STRING database was queried for high confidence (score > 0.7) full interaction networks. All available evidence channels were included to maximize the reliability and completeness of the network. An initial attempt was made to cluster all annotated genes in the genome to identify functional modules. However, due to the limited annotation coverage and species mismatch in STRING, we have chosen to proceed with a targeted approach. Therefore, we used the genes annotated in our genome directly involved in IAA biosynthesis or associated with convergent metabolic pathways, such as those related to tryptophan metabolism [23]. Moreover, we also selected the genes related to mechanisms of salinity adaptation (such as ion transporters, genes involved in osmotic control, etc.) [24]. Furthermore, after comparative evaluation, we found that the QH24 network produced more robust and coherent interaction maps when *H. elongata* was used as the reference species. Conversely, *H. sp. TD01* provided a better match for QH23, showing higher interaction confidence scores and better alignment of orthologous gene contexts. These species were therefore selected as templates for the final interaction network models.

Resulting interaction networks were imported in Cytoscape v3.10.3 [25] for visualization and analysis. The Network Analyzer tool included in Cytoscape was employed using default settings to compute a comprehensive set of network metrics. These included basic parameters such as the number of nodes and edges, network diameter, radius, density, and clustering coefficient. Centrality measures were also calculated, including degree, betweenness centrality, closeness centrality, and stress centrality.

## Results

### Phylogenetic Analysis

**Fig. 1:**
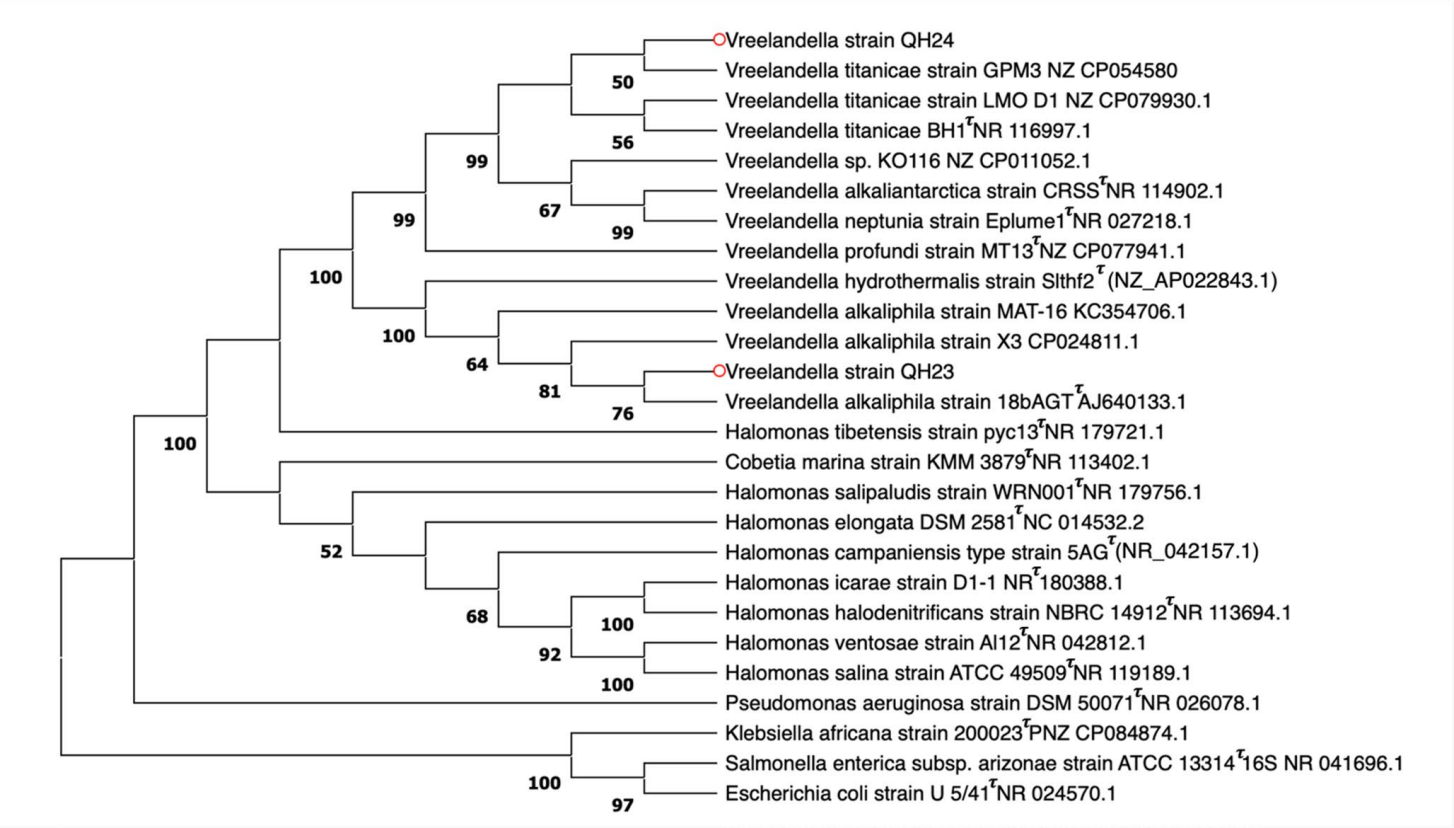
Taxonomical positions of QH24 and QH23 bacterial strains (red circles) based on the 16 S rDNA sequence. Cladogram obtained by the neighbor-joining method, show the relationships of *Vreelandella titanicae* strain QH24 and *Vreelandella alkaliphila* strain QH23 with the related bacterial species. Bootstrap values are reported at the branch points, and nodes with bootstrap values below 50% were considered unresolved and not reliable for inferring phylogenetic relationships. ^τ^indicates the type strains. The access number of the relative strains is indicated

The alignment of the obtained sequences shows an identity of 97.39% between the two strains subject of our studying and their taxonomic collocation into the *Vreelandella* genus (phylum of *γ-Proteobacteria*, family of *Halomonadaceae*). A cladogram based on 16 S rRNA gene sequences (Fig. 1) revealed that QH24 cluster closely with *V. titanicae* strains GPM3 and LMO D1 (99.54% sequence identity), as well as *V. titanicae* BH1^T^, supported by a high bootstrap value of about 99%, indicating strong phylogenetic relation and confirming its placement within the *V. titanicae* clade.

Similarly, QH23 grouped within a well-supported clade (bootstrap value 81%) that includes *Vreelandella alkaliphila* strains 18bAGT^T^ (100% sequence identity), X3, and MAT-16. This clustering is consistent with the close evolutionary relationship of the strain QH23 to *V. alkaliphila* specie.

### Genome Analysis of QH24 and QH23

**Fig. 2:**
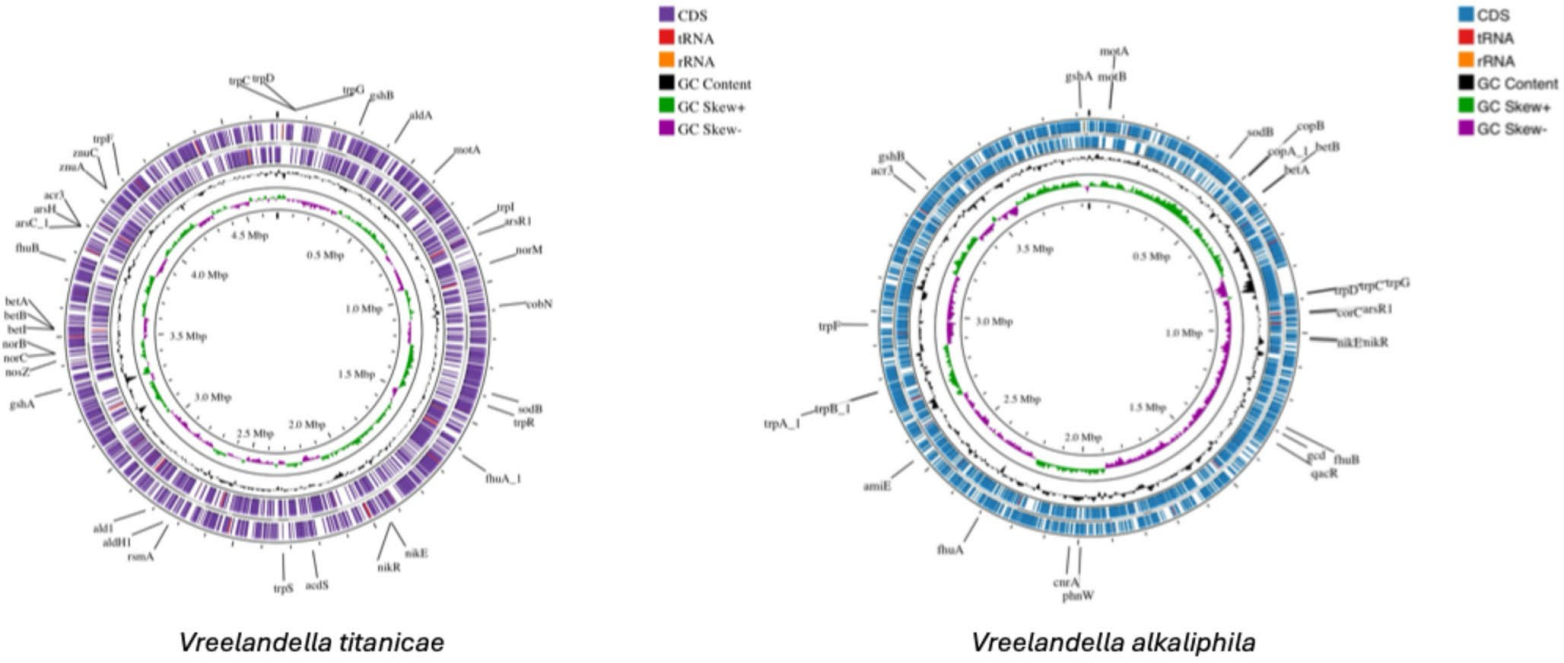
Circular representation of QH24 and QH23 genomes. The two outermost rings represent two strands of the genome, in which are display the functional annotation of genes (CDS), tRNA, and rRNA distribution. The third ring (black color) shows the GC content, while the four ring (green and violet colors) represents the GC skewness throughout the genome (Color figure online)

The genome assembly data of both strains QH24 and QH23 are reported in supplementary materials, Table SM1. In particular, the genome of *V. titanicae* resulted in a circular chromosome (Fig. 2) of approximately 4.7 Mb, with an average G + C content of 54.1%. The genome was processed for gene prediction, and 4.202-coding sequences (CDS) have been annotated, along with genes encoding 59 tRNA and 3 rRNA. The CDS constitute 4.2 Mb (~ 90%) of the genome, with an average gene length of 954 bases. Moreover, the number of COG was 1999, and we recognized 180 MGEs and 3 probable ARGs.

Contrarily, the genome of QH23 was smaller (~ 3.9 Mb), 3582 CDS were annotated (~ 89% of the genome), and we recognized 1759 COG and 103 MGEs. The average length of the genes is comparable to QH24 (~ 967.17), as well as the G + C content (~ 53%) and the presence of 3 probable ARGs. Furthermore, in QH23 were highlighted 4 CRISPR region (vs. only one in QH24).

**Fig. 3:**
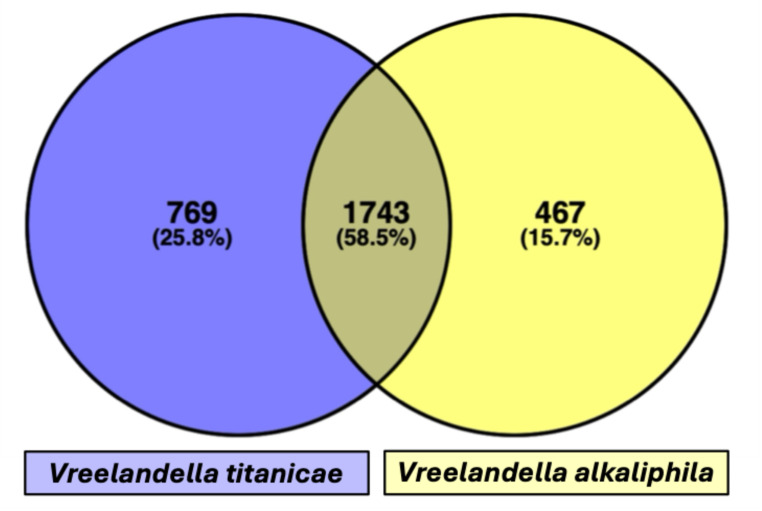
Venn diagram of the shared annotated genes and their percentages. In the middle are reported the genes shared by the two species, in purple those present in QH24, while in yellow those present only in QH23 (Color figure online)

Finally, the shared annotate genes (defined with the same gene name) between the two species were 1743 (58.6%), while 769 genes (25.8%) are present only in QH24, and 467 (15.7%) only in QH23 (Fig. 3).

### Functional Genome Comparison

**Fig. 4:**
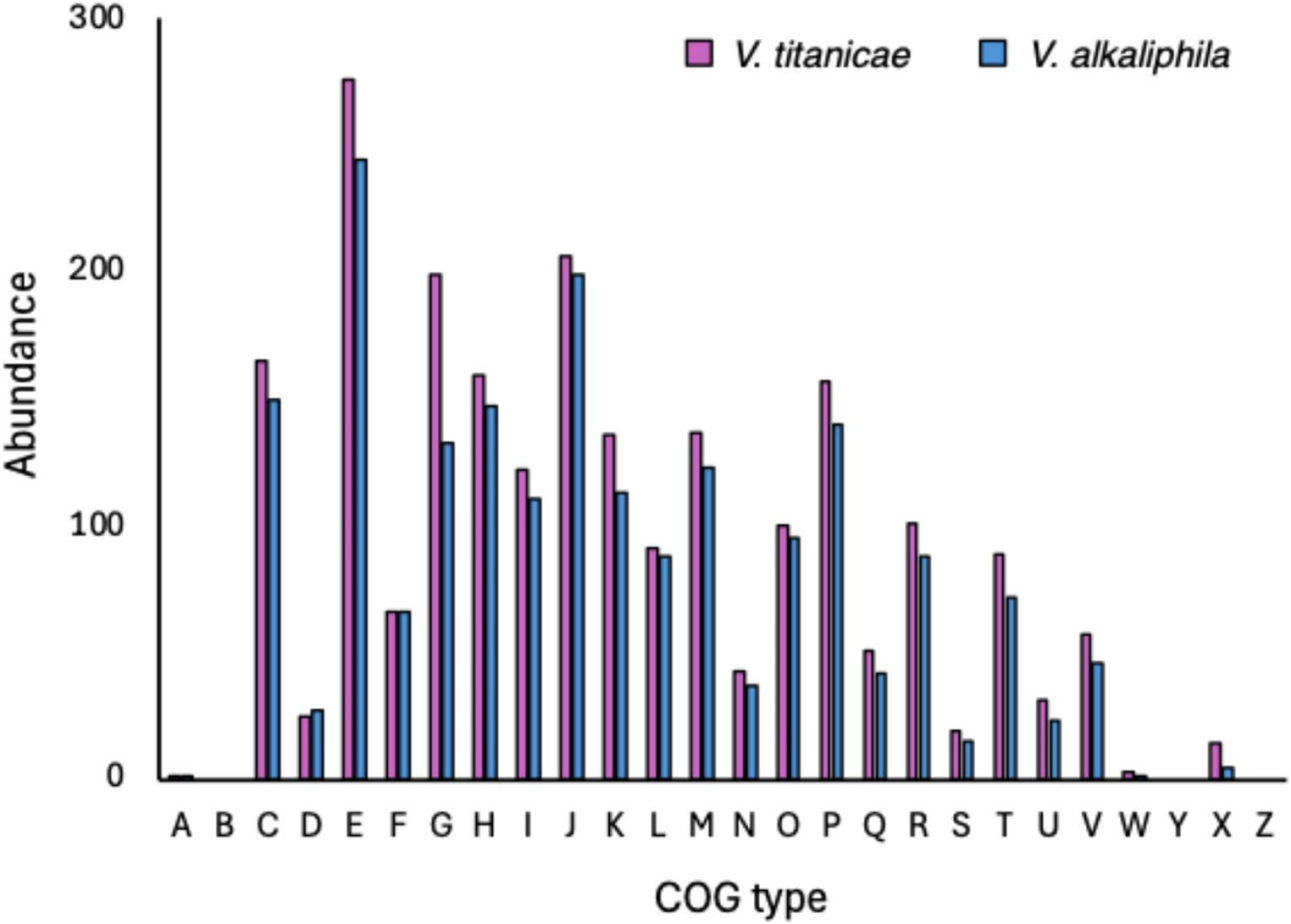
Predicted genes of *V. titanicae* (QH24) and *V. alkaliphila* (QH23) genomes through COG database. (A) RNA processing and modification, (B) Chromatin structure and dynamics, (C) Energy production and conversion, (D) Cell cycle control, cell division, chromosome partitioning, (E) Amino acid transport and metabolism, (F) Nucleotide transport and metabolism, (G) Carbohydrate transport and metabolism, (H) Coenzyme transport and metabolism, (I) Lipid transport and metabolism, (J) Translation, ribosomal structure and biogenesis, K) Transcription, L) Replication, recombination and repair, M) Cell wall/membrane/envelope biogenesis, N) Cell motility, O) Posttranslational modification, protein turnover, chaperones, P) Inorganic ion transport and metabolism, Q) Secondary metabolites biosynthesis, transport and catabolism, R) General function prediction only, S) Function unknown, T) Signal transduction mechanisms, U) Intracellular trafficking, secretion, and vesicular transport, V) Defense mechanisms, W) Extracellular structures, Y) Nuclear structure, X) Function unknown, Z) Cytoskeleton

**Fig. 5:**
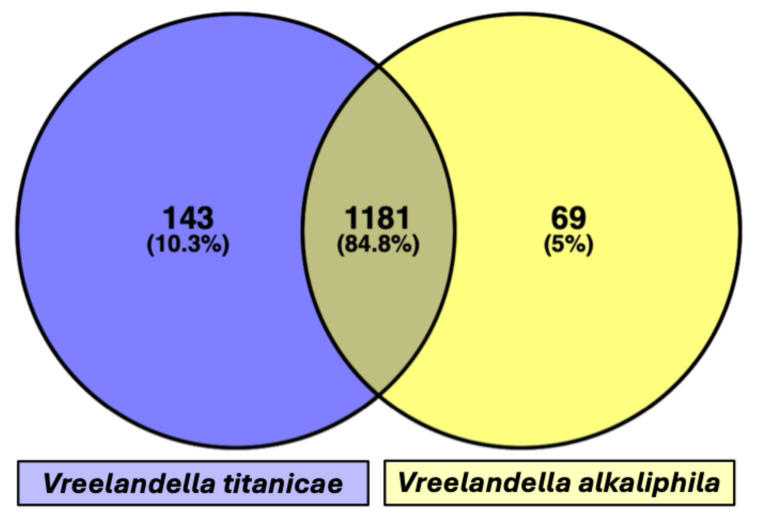
Venn diagram of the shared COG categories as percentage. In the middle are reported the COG shared by the two species, in purple those present in *V. titanicae* (QH24), while in yellow those present only in *V. alkaliphila* (QH23) (Color figure online)

Functional analysis of CDS was carried out using the COG classification. This allowed grouping of genes into standardized functional categories based on orthologs and conserved biological roles (Fig. 4). The results showed significant differences in functional composition between the two species. In *V. titanicae* the most represented COG categories included energy production and conversion (C) 165 genes, amino acid transport and metabolism (E) 276, carbohydrate transport and metabolism (G) 199, transcription (K) 206, defense mechanisms (V) 57, respectively (Fig. 4). In QH23, while maintaining a similar functional distribution in general terms, a relative reduction in gene abundance is observed. However, a greater gene abundance (27 vs. 25 genes) was showed in cell cycle control (D) (Fig. 4). Finally, the shared annotated COG between the two species were 1181 (84.8%), while 143 COG (10.3%) are present only in QH24, and 69 (5.0%) only in QH23 (Fig. 5).

### Identification of Plant Growth Promoting-Related Genes and Genes Associated with Osmotic Stress Response

Halotolerant and halophilic bacteria adopt unique osmoregulation mechanisms to survive in saline environments. Genome analysis of QH24 and QH23 highlighted the presence of several genes involved in osmotic stress tolerance (Tab. SM2) as *pro*S, *opu*E, *bet*ABIT, *gcv*A, *gbs*A, *yeh*WXYZ, and *ect*ABCDP, these latter involved in ectoine biosynthesis.

The genome analysis of QH24 has highlighted many genes involved in plant growth promoting activities (Tab. SM3): *trp*A, *trp*B, *trp*C, *trp*D, *trp*G, *trp*S, and *ald*A that code for enzymes involved in IAA biosynthetic pathway; *fhu*B, *fhu*C, *fhu*F, *yfi*Y, *yfi*Z, *yfh*A and *yus*V, involved in iron transport; *pst*S, *pho*U, *pst*B3, *pho*P, for phosphatase production, and *acd*S for ACC deaminase enzyme. In accordance with our previous work, this bacterium was demonstrated to produce IAA, siderophores, phosphatase, and ACC deaminase [12]. Contrarily, QH23 lacked *ald*A and *acd*S genes, but we observed the presence of *ami*E probably involved in IAA biosynthetic pathway and the genes involved in siderophore, and phosphate transport.

### Plant Growth Promoting Capability

**Fig. 6:**
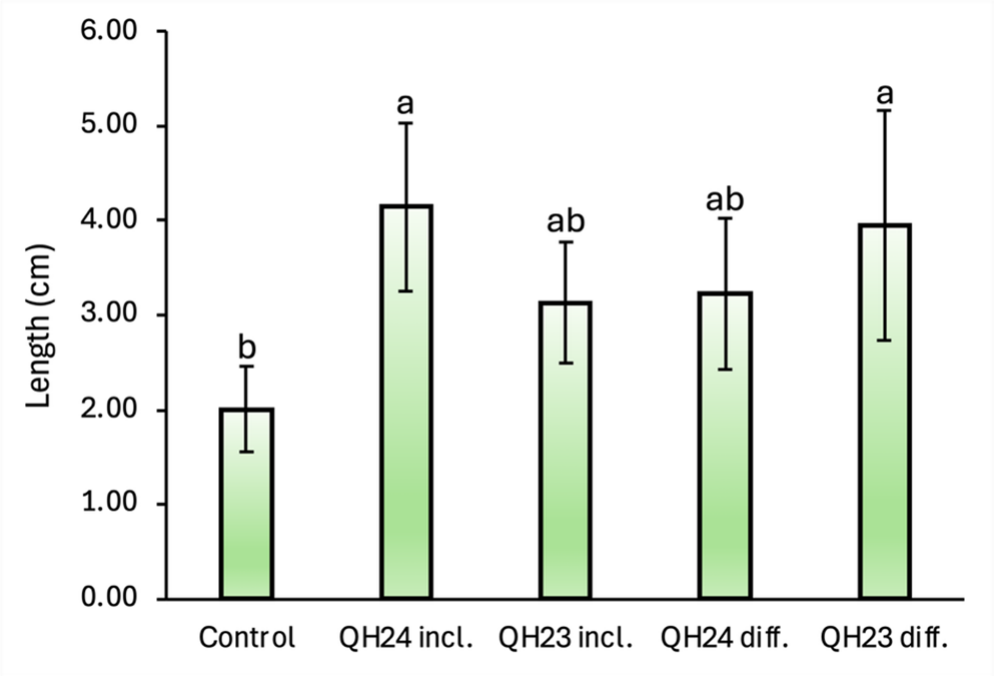
Seedling total length. “incl.” = included; “diff.” = diffused. Letters above the bars indicates a statistically significant difference (ANOVA, Tukey’s post-hoc test, *p* < 0.05)

The PGPR capacity of both strains was tested by evaluating their effect on quinoa seed germination. Total seedling length was significantly influenced by bacterial treatment (Fig. 6, Fig. SM1). Control seedlings exhibited the lowest mean length (2.01 ± 0.46 cm), while inoculation with strain QH24 resulted in the strongest growth promotion effect. In particular, the inclusion method (QH24 incl.) produced the highest mean plant length (4.14 ± 0.90 cm), which was significantly greater than the control (*p* < 0.05). A similarly high mean value was observed for QH23 applied by diffusion (QH23 diff.). Intermediate values were recorded for QH23 inclusion (QH23 incl.) and QH24 diffusion (QH24 diff.). These treatments were not significantly different from either the control or the highest-performing treatments, indicating a moderate but less consistent growth-promoting effect.

**Fig. 7:**
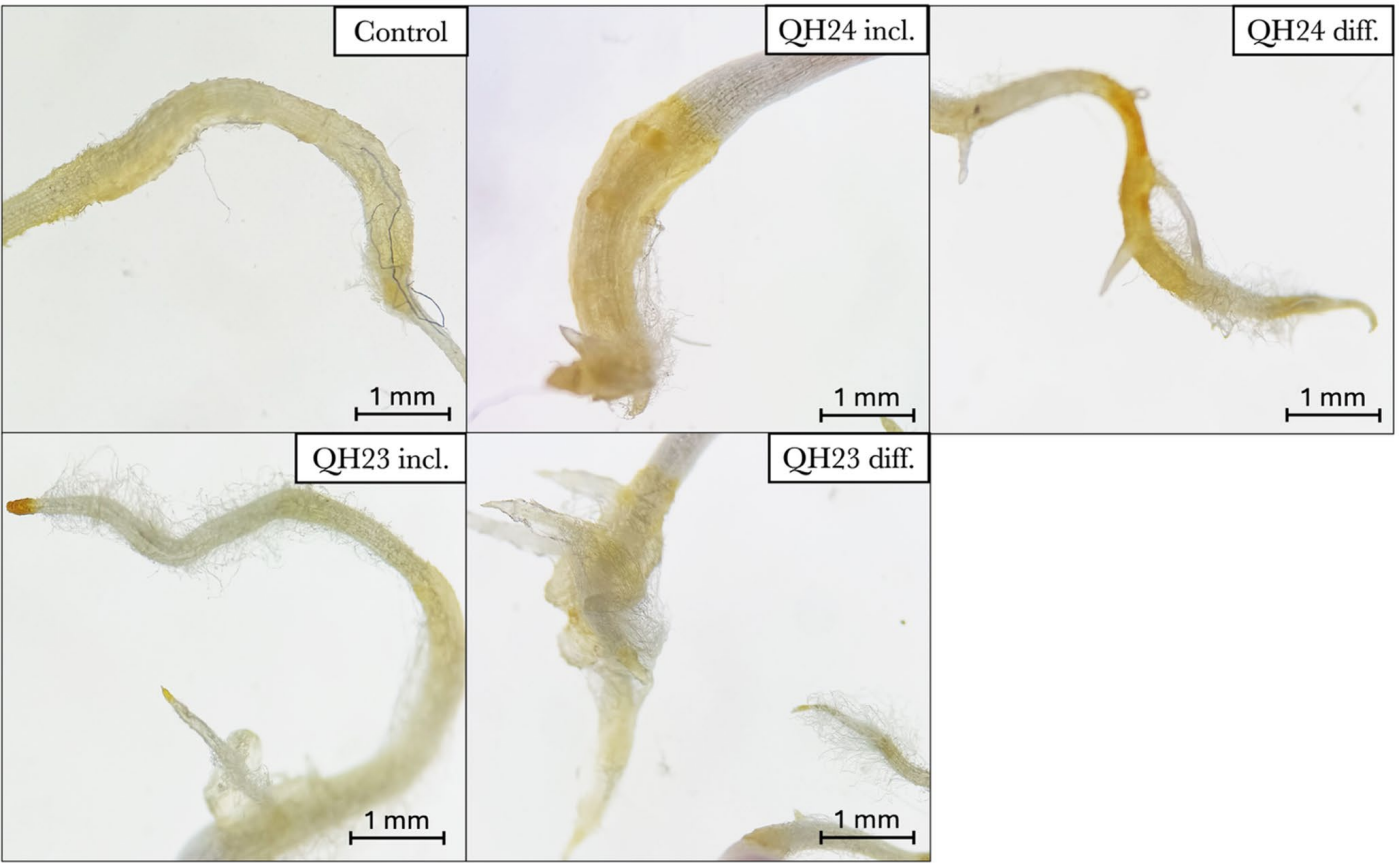
Optical microscopy of the primary root. “incl.” = included; “diff.” = diffused

Microscopic observation of the root systems (Fig. 7) revealed clear morphological differences between control and PGPR-treated seedlings. Control roots exhibited a relatively smooth surface with a limited density of root hairs and minimal development of lateral roots. In contrast, seedlings inoculated with QH24 and QH23 displayed a marked increase in root hair density and improved lateral root formation. This effect was particularly evident in the inclusion treatments, where roots appeared more branched and showed a visibly thicker root hair zone along the elongation region. The diffusion treatments also promoted secondary root emergence compared to the control, although the intensity of the response varied between strains.

### Bacterial Growth and IAA Production in Saline Conditions

**Fig. 8:**
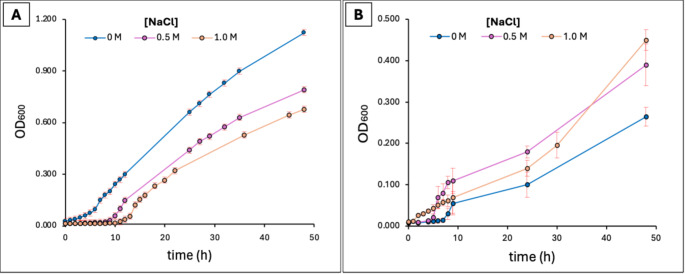
(**A**) Growth curve of *V. titanicae* (QH24) [21] and (**B**) growth curve of *V. alkaliphila* (QH23), at three different NaCl concentration (0, 0.5, 1.0 M)

Growth kinetics were analyzed under the same experimental framework previously validated for IAA production in *Vreelandella titanicae* QH24 [21]. In this strain NaCl concentrations (0, 0.5, 1.0 M) affects different parameters of the growth in modified Marine-Broth. Specifically, the lag phase was shorter in absence of NaCl and longer at highest concentration, < 1.0 h and > 7.0 h, respectively; the cell density at 48 h (beginning of the stationary phase) was greater in absence of salt (1.123 ± 0.001 OD_600_ vs. 0.791 ± 0.011 OD_600_ at 0.5 M, and 0.645 ± 0.009 OD_600_ at 1.0 M NaCl) (Fig. 8A). The average growth rate was comparable across all NaCl concentrations, with values of 0.064 h^− 1^ without NaCl, 0.060 h^− 1^ at 0.5 M, and 0.065 h^− 1^ at 1.0 M NaCl.

In the case of QH23 (Fig. 8B) the baseline growth curves showed a shorter lag phase at 1.0 M NaCl (∼ 2.0 h) compared to other two saline concentrations (< 3.0 h at 0.5 M and > 5.0 h at 0 M). As in the case of QH24, the mean growth rates showing slight variations were comparable across the three growth conditions (0.074 h^− 1^ at 0.M, 0.077 h^− 1^ at 0.5 M, and 0.062 h^− 1^ at 1.0 M NaCl). Finally, the cell density at 48 h (late exponential phase) increased with the presence of NaCl, with the greatest cell amount at 1.0 M (0.45 ± 0.031 OD_600_ at 1.0 M vs. 0.390 ± 0.051 OD_600_ at 0.5 M, and 0.265 ± 0.123 OD_600_ without NaCl).

**Fig. 9:**
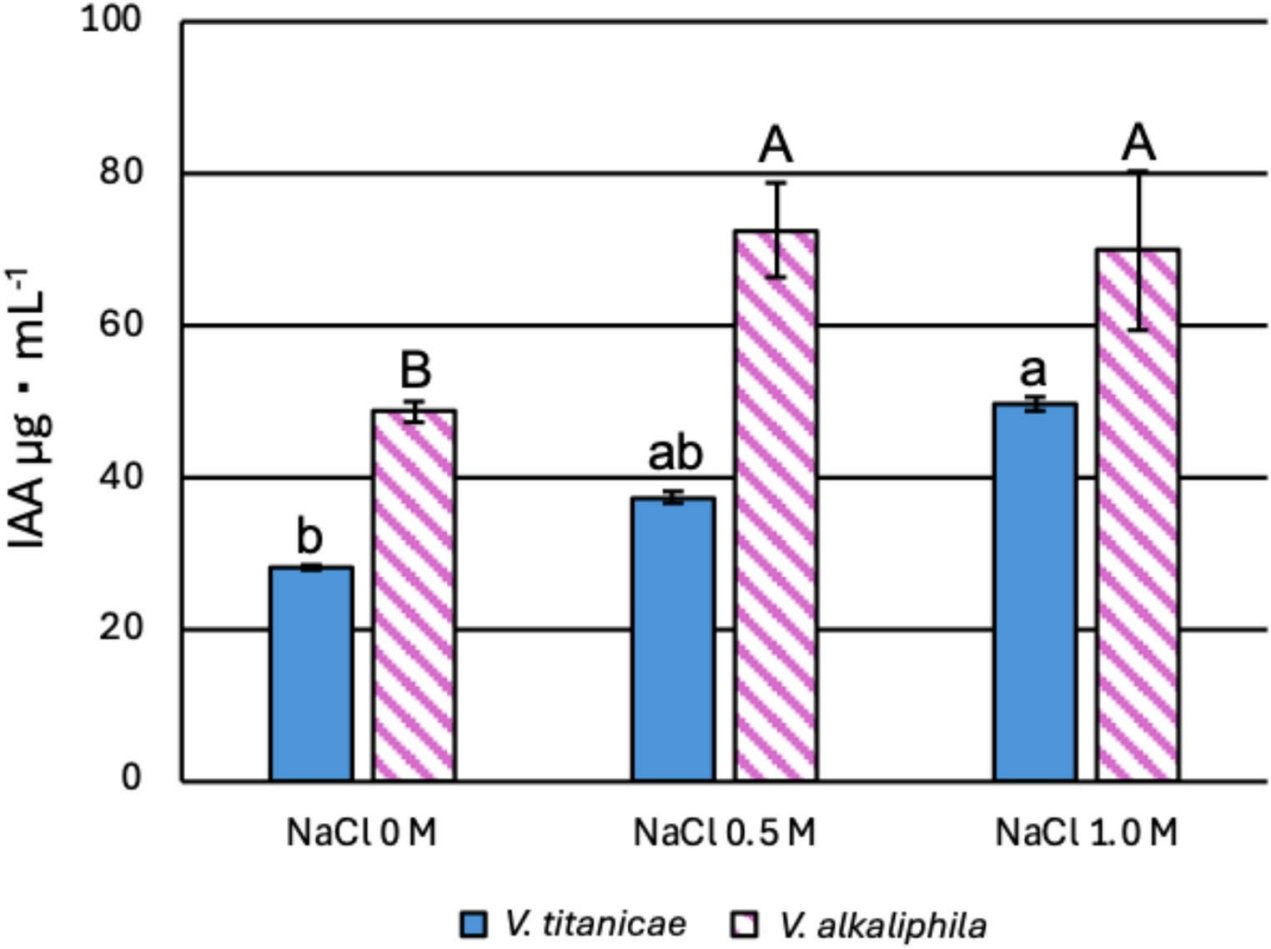
IAA production evaluation for *V. titanicae* (QH24) and *V. alkaliphila* (QH23) at 48 h of incubation and at three different NaCl concentrations (0, 0.5, 1.0 M). Different lowercase letters indicate significant differences among NaCl treatments for QH24, while different uppercase letters indicate significant differences among NaCl treatments for QH23 (ANOVA, Tukey’s post-hoc test, *p* < 0.05)

Afterwards, it was evaluated the IAA production at various saline conditions (Fig. 9). After 48 h of growth, the total IAA production was higher in QH23 compared to QH24 and for both bacteria it was increased by NaCl (48.73 ± 1.29 vs. 28.22 ± 0.27 µg mL^− 1^ at 0 M; 72.6 ± 6.1 vs. 37.4 ± 0.64 µg mL^− 1^ at 0.5 M; 70.00 ± 10.44 vs. 49.78 ± 0.97 µg mL^− 1^ at 1.0 M). In the strain QH24 the total IAA production increased of ~ 25% at 0.5 M and ~ 55% at 1.0 M, compared to bacteria growth without NaCl. Likewise, the strain QH23 exhibited an IAA production increase of ~ 34% at both 0.5 and 1.0 M.

### Gene/Protein Interaction Network Model

**Fig. 10:**
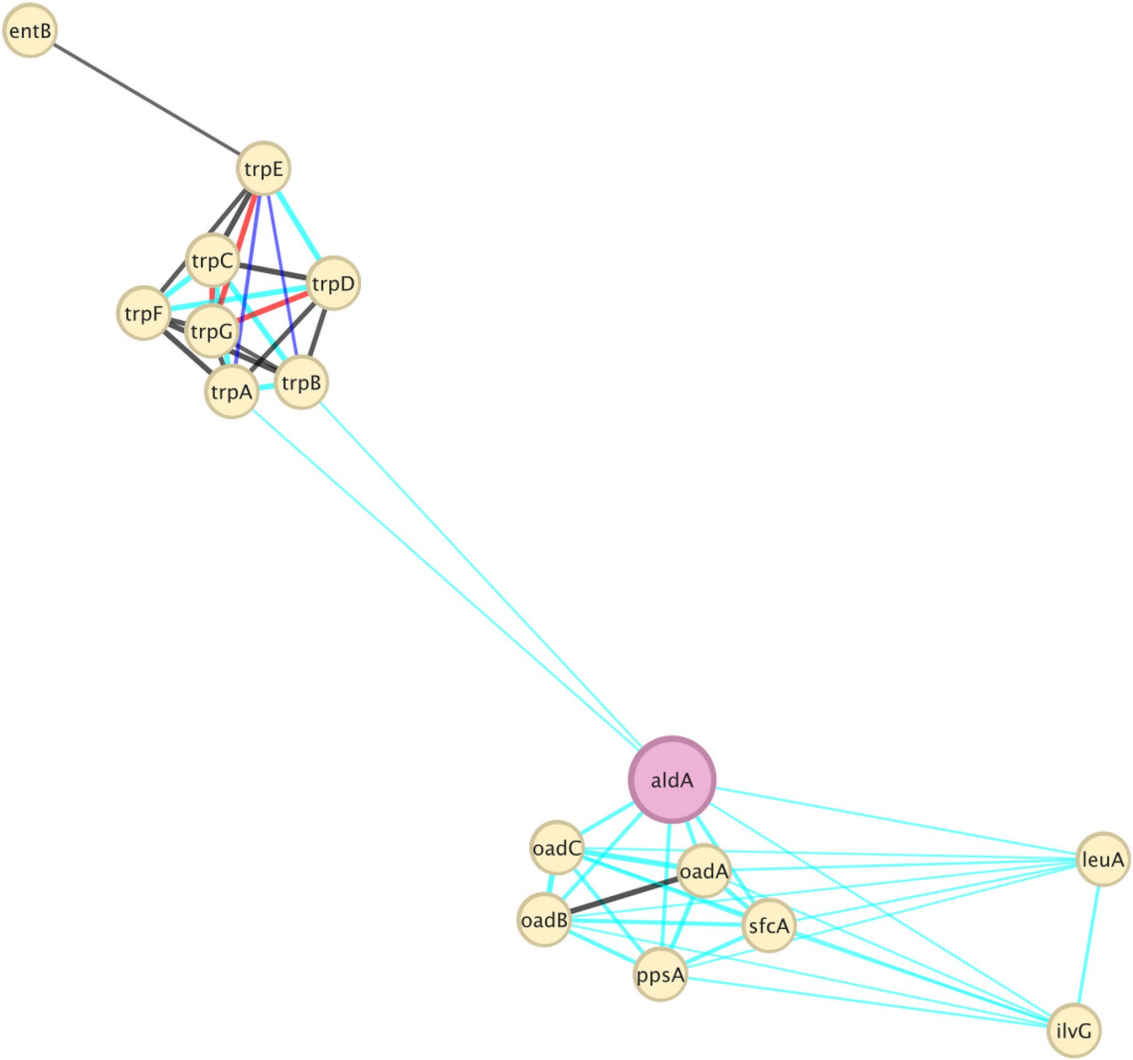
Gene interaction network of *V. titanicae* (QH24), generated in Cytoscape using the STRING database. The edge score is ≥ 0.7; edges colors indicate: black = co-expression; light blue = database-based interactions; red = fusion; blue = cooccurrence (Color figure online)

To investigate the potential regulatory interactions underlying IAA biosynthesis in response to increasing NaCl concentrations, we developed in silico gene/protein interaction networks using the STRING database and visualized them in Cytoscape. Although STRING does not directly simulate environmental stress conditions, protein interaction patterns may offer insights into co-regulated or functionally linked pathways. For QH24 (Fig. 10) we centered the network on *ald*A, a gene involved in the last step of the IAA biosynthetic pathway via indole-3-pyruvate [26]. The network showed a unique interaction cluster with a group of genes related to tryptophan biosynthetic pathway (*trp*A, *trp*B, *trp*C, *trp*D, *trp*E, *trp*F, *trp*G), and another one associated to genes/proteins involved in fundamental metabolism (*oad*A, *oad*B, *oad*C, *sfc*A, *pps*A, *leu*A, *ilv*G). The topological analysis (Tab. SM4) highlighted the central role of *ald*A as functional hub, with the highest degree (9.0), betweenness centrality (0.54), and closeness centrality (0.74), with a clustering coefficient of 0.61.

**Fig. 11:**
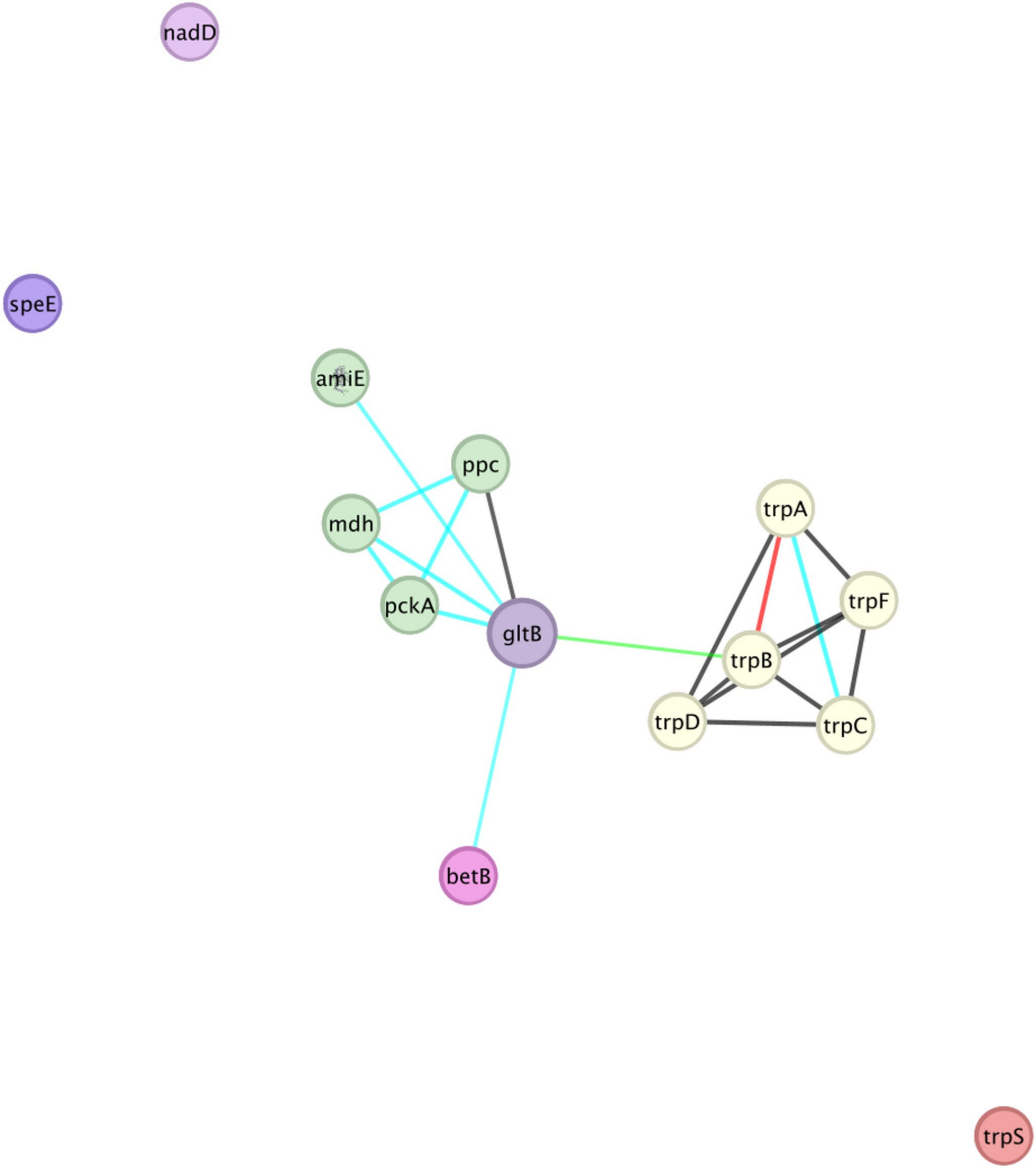
Gene interaction network of *V. alkaliphila* (QH23), generated in Cytoscape using the STRING database. The edge score is ≥ 0.7; edges colors indicate: black = co-expression; light blue = database-based interactions; red = fusion; green = neighborhood. Green nodes = first cluster; yellow nodes = second cluster (Color figure online)

The refined protein interaction network centered on *ami*E in QH23 (Fig. 11) revealed a more complex and structured set of interactions than previously indicated in the case of QH24. Topological analysis (Tab. SM5) showed that *ami*E has a degree of 7.0, a betweenness centrality of 0.31, closeness centrality of 0.62, and a clustering coefficient of 0.33.

## Discussion

The results illustrated in our study provide new information about genomic and functional diversity of two bacterial strains belonging to the *Vreelandella* genus: QH24 and QH23, highlighting their potentiality as PGPR.

The two bacterial strains share about 84.8% and 58.5% of the total annotated COG and CDS, respectively. The comparative genome analysis showed that the two strains may adopt similar mechanisms to survive in saline environments. In fact, we highlighted the presence, in both of them, of several genes involved in osmoregulation specifically found in *Vreelandella* genera, as *ect*, *pro*, *bet*, *yeh*, etc., being involved in the synthesis, uptake and transport of compatible solutes [27]. Previous studies support our findings, for example [28, 29] it was observed the presence of *ect*ABC and *bet*AB genes in *Vreelandella sp.*, responsible for the biosynthesis of ectoine and glycine betaine, respectively. Similarly, Sabroso et al. (2026) [30] observed *pro*ABC genes in *Vreelandella titanicae Zn11_249*, that mediates proline biosynthesis under osmotic stress. These genes encode key enzymes for the production and accumulation of osmoprotectant such as proline, ectoine, and glycine betaine that do not interfere with cellular metabolism and serve to balance the osmotic pressure between the cytoplasm and the external saline environment. The accumulation of these compatible solutes is a fundamental strategy for halophilic and halotolerant microorganisms to prevent cellular dehydration, protect protein structure and function, and maintain ionic homeostasis [31]. Moreover, a part of these genes is involved in the uptake of exogenous osmoprotectant, suggesting that these strains may utilize both de novo synthesis and molecules to adapt to environmental osmotic stress. This dual strategy may confer greater flexibility and energy efficiency under fluctuating saline conditions.

Despite the identification of numerous shared genes involved in osmotic stress tolerance, both strains exhibited different physiological behaviors under saline conditions. The growth kinetics evidenced the slighter halophilic nature of QH24 compared to QH23. In both strains, NaCl mainly affected lag phase duration and biomass yield rather than the mean exponential growth rate. QH24 showed higher final biomass and reduced lag phase in the absence of additional NaCl. In contrast, QH23 reach the highest cell density and exhibit a shorter lag phase at 1.0 M NaCl. This difference between the two strains is coherent with the genomic features of osmoadaptive identified genes, including compatible solute biosynthesis and transport systems. The enhanced performance of QH23 under higher saline conditions may reflect a more efficient regulation of these osmoprotective mechanisms, allowing better maintenance of cellular homeostasis and metabolic activity in high-salt environments. It should be noted that we describe the strain dependence on NaCl for the optimal growth. In particular, QH24 showed optimal growth in absence of NaCl, more consistent with a halotolerant profile, whereas QH23 did not exhibit a strict salt-dependence, but growth performance improved in presence of NaCl, reflecting a behavior compatible with a moderate halophily rather than strict halophilic. However, considering that the growth experiments were performed in a medium containing significant concentration of other salts in its basal composition, a further characterization by means of a salt gradient test in low-salt culture media should be done for a more accurate classification of the two strains in the halophilic subclasses (slight, moderate, and strictly).

Moreover, genome analysis confirmed the presence of several genes typically associated with PGPR, including those involved in IAA biosynthesis. These genomic traits are in accord with the phenotypic effects observed in our in vitro experiment. We observed morphological changes in quinoa seedlings grown in the presence of QH24 or QH23 highlighting the growth-promoting role of these bacteria. Specifically, we reported an increase in seedling elongation, a greater number of secondary roots, and an increase in root hair density. These effects are commonly associated with IAA, a phytohormone that regulates the root architecture system and stimulates the formation of root hairs and secondary roots [32]. Furthermore, previous studies have showed that IAA-producing bacteria as *Serratia marcescens* and *Azospirillum baldaniorum* increased the number of lateral root and root hairs in *Arabidopsis thaliana* (L.) [33, 34], supporting that the IAA produced by our bacteria may contribute to the modulation of plant root development.

The exclusive presence of *ald*A in QH24 and *ami*E in QH23 could also indicate differences in the mechanisms of IAA biosynthesis. About that, McClerklin et al. (2018) [35] showed that *Pseudomonas syringae* strain DC3000 produced IAA, identifying the indole-3-acetaldehyde dehydrogenase (*ald*A) enzyme had a key role in IAA biosynthesis, catalyzing the NAD-dependent formation of IAA from indole-3-acetaldehyde (IAAld), supporting the indole-3-pyruvate pathway (IPyA). Instead, Grossi et al. (2020) [36] described another IAA biosynthetic pathway in *Methylobacterium* sp. 2 A, where IAA formation was related to conversion of indole-3-acetamide (IAM) into IAA by the aliphatic amidase *ami*E. Although the IPyA pathway is frequently reported among plant-associated bacteria, the IAM way is also well documented and can contribute substantially to auxin biosynthesis. About that, we suppose that QH24 and QH23 could produce IAA using different biosynthetic pathways. Consistent with this complexity, we observed that QH23 produced higher IAA than QH24 despite lacking *ald*A. This suggests that the presence of a specific biosynthetic gene doesn’t necessarily correlate with higher IAA production, since pathway efficiency depends on regulatory control, cofactor availability, and integration with central metabolism. Therefore, the regulatory system could determine the overall IAA production capacity rather than the presence of a single gene (*ald*A or *ami*E).

Moreover, genome analysis highlighted the presence of *acd*S gene in QH24 and not in QH23. This observation was confirmed by our previous work where we described the main PGP feature of these strains, quantifying the ACC deaminase activity of QH24 [12].

Commonly, osmotic stress induced by NaCl would lead to reduced IAA production by microorganisms [37]. However, in our case, the quantification of IAA amount in the culture medium showed that both bacterial strains produce a greater quantity of this hormone in the presence of NaCl, with higher levels in QH23 than QH24. Some studies have reported an increase in IAA production in the presence of NaCl in the culture media, but its regulation remained unknown. Sharma et al. (2023) [38] observed an increase in IAA biosynthesis according to the NaCl concentrations in *Virgibacillus halodenitrificans* ASH15, (54.79 ± 1.80 µg mL^− 1^ vs. 48.73 ± 1.29 µg mL^− 1^ without NaCl, and 76.19 ± 2.40 µg mL^− 1^ vs. 70.00 ± 10.44 µg mL^− 1^ at 1.0 M NaCl, respectively). Moreover, Yousef et al. (2018) [39] observed an increase of IAA production in *Bacillus subtilis* CW-2 in relation to NaCl amounts added culture medium, albeit adopting less severe saline conditions (0.1–0.2 M), without discuss the mechanism regulation.

The gene *ald*A exhibited the most direct interactions suggesting its role in linking genes involved in tryptophan biosynthesis with those related to fundamental metabolism. Its betweenness centrality is the highest among all nodes indicating that *ald*A acts as a bridge connecting distinct functional clusters. Moreover, its relatively high closeness centrality suggested that it could rapidly interact with other nodes in the network, potentially enabling tight coordination between metabolic and regulatory processes. This is consistent with the hypothesis that *ald*A, beyond catalyzing the oxidation of IAAld to IAA, may act as an integrative node between IAA biosynthesis and salt-responsive metabolic pathways. Specifically, the gene *ald*A can be considered a metabolic hub that directly couples salt-driven energy metabolism to auxin production [35]. At high NaCl concentrations, activation of the Na⁺ oxaloacetate decarboxylase complex (*oad*ABC) enhances oxaloacetate decarboxylation and concomitant Na⁺ translocation, regenerating NAD⁺ and sustaining tricarboxylic acid (TCA) cycle flux [40]. We hypothesized that the increase in NAD⁺ availability could enforce *ald*A activity mediating oxidation of IAAld to IAA, thereby stimulating auxin biosynthesis. Concurrent upregulation of ectoine and glycine-betaine biosynthetic operons stabilizes cytoplasmic and enzymatic proteins and membranes, preserving their structure and function in hyperosmotic conditions [7]. Transcriptional regulators sensitive to intracellular osmolarity (e.g.., *bet*I or *opu*E) may further coordinate expression of both trp-operon genes and *ald*A, ensuring a steady supply of indole precursors [23, 37].

In the case of QH23, the gene *ami*E plays a moderately central role within the network, acting not only as a connector between various metabolic functions, but also participating in a partially clustered module of functionally related genes. Functionally, *ami*E is linked to key enzymes in central carbon metabolism (*ppc*, *pck*A, *mdh*) [41], as well as to *bet*B, involved in the synthesis of glycine betaine [42]. Therefore, the gene *ami*E may be involved in coordinating IAA biosynthesis (via the IAM pathway) with osmotic stress responses. The intermediate values of betweenness and closeness centrality support the idea that *ami*E functions as a mediator node, bridging distinct yet related biological processes, without acting as the primary hub. Specifically, QH23 employs the IAM pathway, wherein the aliphatic amidase *ami*E hydrolyzes IAM to IAA independently of NAD⁺ [23]. The *ami*E gene interacts with central carbon metabolism enzymes that govern the balance between gluconeogenesis and TCA cycle activity. Salt-induced accumulation of glycine-betaine via *bet*AB and *yeh*WXYZ transporters maintains cytoplasmic pH and membrane potential, critical conditions for optimal *ami*E catalytic activity [7]. Moreover, the glutamate synthase *glt*B node suggests a feedback mechanism in which enhanced ammonium assimilation and glutamate production modulate IAM availability and thus IAA production [36].

## Conclusion

The comparative genomic analysis of the two *Vreelandella* strains (QH24 and QH23) revealed key differences in their potential as plant growth-promoting rhizobacteria (PGPR), particularly in the biosynthesis and regulation of IAA. Both strains exhibit genetic traits associated with osmotic stress tolerance and siderophore production, while their divergence in IAA biosynthesis pathways suggests distinct strategies for modulating plant growth. Our comparative genomic and in silico network analyses allow to identify the potential regulatory hubs and to hypothesize how these bacterial strains differently coordinate IAA biosynthesis with osmoadaptation, guiding targeted experiments in a cost-effective manner. However, without transcriptional profiling, we cannot definitively confirm whether key genes (e.g., *ald*A, *ami*E, *oad*ABC, *bet* and *ect* operons) are differentially expressed under salt stress. Likewise, metabolomic analysis is necessary to quantify intracellular and extracellular levels of NAD⁺/NADH, IAAld, IAM, IAA, and glycine‐betaine to validate the proposed fluxes. All these studies are being carried out in our laboratory to give a clearer picture of the IAA biosynthesis and production by QH24 and QH23.

## Electronic Supplementary Material

Below is the link to the electronic supplementary material.

Supplementary Material 1

## Data Availability

The genomes of the two bacterial strains descripted in this work were submitted to NCBI and are publicly available under projects PRJNA1322357 (QH24 strain) and PRJNA1322382 (QH23 strain). Any raw data files be needed in another format they are available from the corresponding author upon reasonable request.
