# Supplementary material for: Genomic and Functional Analysis of Two Halophilic IAA-Producing Vreelandella Strains
Source: Curr Microbiol. 2026 May 19;83(7):368. doi: 10.1007/s00284-026-04946-7 (PMC13186816; doi:10.1007/s00284-026-04946-7)
Supplement: Supplementary file 1 — Supplementary Material 1 [file 284_2026_4946_MOESM1_ESM.docx]

**Supplementary materials**

Journal: **Current Microbiology**

**Genomic and Functional Analysis of Two Halophilic IAA-Producing *Vreelandella* Strains**

Gianmaria Oliva^1^, Bruno Hay Mele^2^, Concetta Di Lorenzo^3^, Mimmo Turano^3^, Stefano Castiglione^1*^, Giovanni Vigliotta^1^

^1^ Department of Chemistry and Biology “A. Zambelli”, University of Salerno, 84084 Fisciano (SA), Italy

^2^ Department for the Promotion of Human Science and Quality of Life, San Raffaele Open University, 00166, Rome (RM), Italy

^3^ Department of Biology, University of Naples Federico II, 80126, Naples (NA), Italy

^*^ Corresponding author

*E-mail address*: [scastiglione@unisa.it](mailto:scastiglione@unisa.it)

**Table SM1**. Genome characteristics of *V. titanicae* (QH24) and *V. alkaliphila* (QH23)

|  | QH24 | QH23 |
| --- | --- | --- |
| Characteristics | Values | |
| Genome size (bp) | 4692021 | 3936074 |
| Chromosome | 1 | 1 |
| Chromosome size (bp) | 4692021 | 3936074 |
| GC content (%) | 55.14 | 52.56 |
| Topology | Circular | Circular |
| tRNA | 59 | 56 |
| rRNA (5S, 16S, 23S) | 3 | 2 |
| CDS | 4372 | 3582 |
| CDS (bp) | 4223638 | 3522435 |
| CDS (% genome) | 90.01 | 89.50 |
| Average gene length (bp) | 952.90 | 967.17 |
| CRISPR | 1 | 4 |
| Antibiotic resistance genes | 3 | 3 |
| Mobile genetic elements | 180 | 103 |
| Genes annotated with COG | 1999 | 1759 |

**Table SM2.** Genes predicted to be involved in abiotic stress response

| Osmotic stress | Gene | EC | COG | Product | |
| --- | --- | --- | --- | --- | --- |
| *Vreelandella titanicae* (QH24) | | | | | |
|  | *opu*E |  | COG0591 | Osmoregulated proline transporter OpuE | |
|  | *pip* | 3.4.11.5 |  | Proline iminopeptidase | |
|  | *pro*S | 6.1.1.15 | COG0442 | Proline--tRNA ligase | |
|  | *doe*A | 3.5.4.44 | COG0006 | Ectoine hydrolase | |
|  | *ect*A | 2.3.1.178 |  | L-2,4-diaminobutyric acid acetyltransferase | |
|  | *ect*B | 2.6.1.76 | COG0160 | Diaminobutyrate--2-oxoglutarate transaminase | |
|  | *ect*C | 4.2.1.108 |  | L-ectoine synthase | |
| Ectoine | *ect*D | 1.14.11.55 |  | Ectoine dioxygenase | |
|  | *ect*P |  | COG1292 | Ectoine/glycine betaine/proline transporter EctP | |
|  | *tea*A |  | COG1638 | Ectoine-binding periplasmic protein TeaA | |
|  | *tea*B |  | COG3090 | Ectoine TRAP transporter small permease protein TeaB | |
|  | *tea*C |  | COG1593 | Ectoine TRAP transporter large permease protein TeaC | |
|  | *ueh*B |  | COG3090 | Ectoine/5-hydroxyectoine TRAP transporter small permease protein UehB | |
|  | *bet*A | 1.1.99.1 | COG2303 | Oxygen-dependent choline dehydrogenase | |
|  | *bet*B | 1.2.1.8 | COG1012 | NAD/NADP-dependent betaine aldehyde dehydrogenase | |
|  | *bet*I |  | COG1309 | HTH-type transcriptional regulator BetI | |
|  | *bet*T |  | COG1292 | High-affinity choline transport protein | |
|  | *gbs*A | 1.2.1.8 | COG1012 | Betaine aldehyde dehydrogenase | |
|  | *gcv*A |  |  | Glycine cleavage system transcriptional activator | |
| Glycine/  Betaine | *gcv*H |  | COG0509 | Glycine cleavage system H protein | |
|  | *gcv*P | 1.4.4.2 | COG0403 | Glycine dehydrogenase (decarboxylating) | |
|  | *gly*Q | 6.1.1.14 | COG0752 | Glycine--tRNA ligase alpha subunit | |
|  | *gly*S | 6.1.1.14 | COG0751 | Glycine--tRNA ligase beta subunit | |
|  | *opu*AA | 7.6.2.9 | COG4175 | Glycine betaine transport ATP-binding protein OpuAA | |
|  | *opu*AB |  | COG4176 | Glycine betaine transport system permease protein OpuAB | |
|  | *thi*O | 1.4.3.19 |  | Glycine oxidase | |
|  | *yeh*W |  | COG1174 | Glycine betaine uptake system permease protein YehW | |
|  | *yeh*X | 3.6.3.- | COG1125 | Glycine betaine uptake system ATP-binding protein YehX | |
|  | *yeh*Y |  | COG1174 | Glycine betaine uptake system permease protein YehY | |
|  | *yeh*Z |  | COG1732 | Glycine betaine-binding protein YehZ | |
| *Vreelandella alkaliphila* (QH23) | | | | | |
|  | *opu*E |  | COG0591 | | Osmoregulated proline transporter OpuE |
| Proline | *pro*S | 6.1.1.15 | COG0442 | | Proline--tRNA ligase |
|  | *stc*D | 1.-.-.- |  | | putative N-methylproline demethylase |
|  | *doe*A | 3.5.4.44 | COG0006 | | Ectoine hydrolase |
|  | *ect*A | 2.3.1.178 |  | | L-2,4-diaminobutyric acid acetyltransferase |
|  | *ect*B | 2.6.1.76 | COG0160 | | Diaminobutyrate--2-oxoglutarate transaminase |
|  | *ect*C | 4.2.1.108 |  | | L-ectoine synthase |
|  | *ect*D | 1.14.11.55 |  | | Ectoine dioxygenase |
| Ectoine | *ect*P |  | COG1292 | | Ectoine/glycine betaine/proline transporter EctP |
|  | *ect*T |  |  | | Ectoine/hydroxyectoine transporter |
|  | *tea*A |  | COG1638 | | Ectoine-binding periplasmic protein TeaA |
|  | *tea*B |  | COG3090 | | Ectoine TRAP transporter small permease protein TeaB |
|  | *tea*C |  | COG1593 | | Ectoine TRAP transporter large permease protein TeaC |
|  | *ueh*B |  | COG3090 | | Ectoine/5-hydroxyectoine TRAP transporter small permease protein UehB |
|  | *bet*A | 1.1.99.1 | COG2303 | | Oxygen-dependent choline dehydrogenase |
|  | *bet*B | 1.2.1.8 | COG1012 | | NAD/NADP-dependent betaine aldehyde dehydrogenase |
|  | *bet*I |  | COG1309 | | HTH-type transcriptional regulator BetI |
|  | *bet*T |  | COG1292 | | High-affinity choline transport protein |
|  | *gbs*A | 1.2.1.8 | COG1012 | | Betaine aldehyde dehydrogenase |
|  | *gcv*A |  |  | | Glycine cleavage system transcriptional activator |
|  | *gcv*H |  | COG0509 | | Glycine cleavage system H protein |
| Glycine/  Betaine | *gcv*P | 1.4.4.2 | COG0403 | | Glycine dehydrogenase (decarboxylating) |
|  | *gly*Q | 6.1.1.14 | COG0752 | | Glycine--tRNA ligase alpha subunit |
|  | *gly*S | 6.1.1.14 | COG0751 | | Glycine--tRNA ligase beta subunit |
|  | *opu*AA | 7.6.2.9 | COG4175 | | Glycine betaine transport ATP-binding protein OpuAA |
|  | *opu*AB |  | COG4176 | | Glycine betaine transport system permease protein OpuAB |
|  | *thi*O | 1.4.3.19 |  | | Glycine oxidase |
|  | *yeh*W |  | COG1174 | | Glycine betaine uptake system permease protein YehW |
|  | *yeh*X | 3.6.3.- | COG1125 | | Glycine betaine uptake system ATP-binding protein YehX |
|  | *yeh*Y |  | COG1174 | | Glycine betaine uptake system permease protein YehY |
|  | *yeh*Z |  | COG1732 | | Glycine betaine-binding protein YehZ |

**Table SM3**. Genes predicted to be involved in plant growth promoting traits

| PGPR activities | Gene | EC | COG | Product |
| --- | --- | --- | --- | --- |
| *Vreelandella titanicae* (QH24) | | | | |
|  | *ald*A | 1.2.1.3 |  | Putative aldehyde dehydrogenase AldA |
|  | *trpA* | 4.2.1.20 | COG0159 | Tryptophan synthase alpha chain |
|  | *trpB* | 4.2.1.20 | COG0133 | Tryptophan synthase beta chain |
| IAA biosynthesis | *trp*C | 4.1.1.48 |  | Indole-3-glycerol phosphate synthase |
|  | *trp*D | 2.4.2.18 | COG0547 | Anthranilate phosphoribosyltransferase |
|  | *trp*G | 4.1.3.27 | COG0512 | Anthranilate synthase component 2 |
|  | *trp*S | 6.1.1.2 | COG0180 | Tryptophan--tRNA ligase |
|  | *fhu*B |  | COG0609 | Iron(3+)-hydroxamate import system permease protein FhuB |
|  | *fhu*C | 7.2.2.16 | COG1120 | Iron(3+)-hydroxamate import ATP-binding protein FhuC |
|  | *fhu*F |  | COG4114 | Ferric iron reductase protein FhuF |
| Siderophores | *yfi*Y |  | COG0614 | putative siderophore-binding lipoprotein YfiY |
|  | *yfi*Z |  | COG0609 | putative siderophore transport system permease protein YfiZ |
|  | *yfh*A |  | COG0609 | putative siderophore transport system permease protein YfhA |
|  | *yus*V |  | COG1120 | putative siderophore transport system ATP-binding protein YusV |
|  | *pst*S |  | COG0226 | Phosphate-binding protein PstS |
| Phosphate metabolism | *pho*U |  | COG0704 | Phosphate-specific transport system accessory protein PhoU |
|  | *pst*B3 | 7.3.2.1 | COG1117 | Phosphate import ATP-binding protein PstB 3 |
|  | *pho*P |  | COG0745 | Alkaline phosphatase synthesis transcriptional regulatory protein PhoP |
| Ethylene stress reduction | *acd*S | 3.5.99.7 |  | 1-aminocyclopropane-1-carboxylate deaminase |
| *Vreelandella alkaliphila* (QH23) | | | | |
|  | *ami*E | 3.5.1.4 |  | Aliphatic amidase |
|  | *trpA* | 4.2.1.20 | COG0159 | Tryptophan synthase alpha chain |
|  | *trpB* | 4.2.1.20 | COG0133 | Tryptophan synthase beta chain |
| IAA biosynthesis | *trp*C | 4.1.1.48 |  | Indole-3-glycerol phosphate synthase |
|  | *trp*D | 2.4.2.18 | COG0547 | Anthranilate phosphoribosyltransferase |
|  | *trp*G | 4.1.3.27 | COG0512 | Anthranilate synthase component 2 |
|  | *trp*S | 6.1.1.2 | COG0180 | Tryptophan--tRNA ligase |
|  | *yfi*Z |  | COG0609 | putative siderophore transport system permease protein YfiZ |
|  | *yfh*A |  | COG0609 | putative siderophore transport system permease protein YfhA |
|  | *yfi*Y |  | COG0614 | putative siderophore-binding lipoprotein YfiY |
| Siderophores | *fhu*A |  | COG1629 | Ferrichrome outer membrane transporter/phage receptor |
|  | *fhu*B |  | COG0609 | Iron(3+)-hydroxamate import system permease protein FhuB |
|  | *fhu*C | 7.2.2.16 | COG1120 | Iron(3+)-hydroxamate import ATP-binding protein FhuC |
|  | *fhu*D |  | COG0614 | Iron(3+)-hydroxamate-binding protein FhuD |
|  | *pst*S |  | COG0226 | Phosphate-binding protein PstS |
| Phosphate metabolism | *pst*B3 | 7.3.2.1 | COG1117 | Phosphate import ATP-binding protein PstB 3 |
|  | *pho*U |  | COG0704 | Phosphate-specific transport system accessory protein PhoU |

**Figure SM1**. Seedling total length. “incl.” = included; “diff.” = diffused.

**Table SM4.** Topological comparison of *ald*A and first neighbour genes in *Vreelandella titanicae* strain QH24

| **Gene** | **Degree** | **Betweenness**  **Centrality** | **Closeness**  **Centrality** | **Clustering**  **Coefficient** |
| --- | --- | --- | --- | --- |
| trpB | 7 | 0.22 | 0.67 | 0.76 |
| trpA | 7 | 0.22 | 0.67 | 0.76 |
| trpG | 6 | 0.0 | 0.48 | 1.0 |
| trpE | 6 | 0.0 | 0.48 | 1.0 |
| trpF | 6 | 0.0 | 0.48 | 1.0 |
| leuA | 7 | 0.0 | 0.54 | 1.0 |
| sfcA | 7 | 0.0 | 0.54 | 1.0 |
| ppsA | 7 | 0.0 | 0.54 | 1.0 |
| oadA | 7 | 0.0 | 0.54 | 1.0 |
| aldA | 9 | 0.54 | 0.74 | 0.61 |
| oadB | 7 | 0.0 | 0.54 | 1.0 |
| oadC | 7 | 0.0 | 0.54 | 1.0 |
| ilvG | 7 | 0.0 | 0.54 | 1.0 |
| trpC | 6 | 0.0 | 0.48 | 1.0 |
| trpD | 6 | 0.0 | 0.48 | 1.0 |

**Table SM5.** Topological comparison of *ami*E and first neighbor genes in *Vreelandella alkaliphila* strain QH23

| **Gene** | **Degree** | **Betweenness**  **Centrality** | **Closeness**  **Centrality** | **Clustering**  **Coefficient** |
| --- | --- | --- | --- | --- |
| trpS | 2 | 0.0 | 0.39 | 1.0 |
| trpA | 5 | 0.026 | 0.56 | 0.9 |
| trpC | 6 | 0.096 | 0.59 | 0.66 |
| trpF | 4 | 0.0 | 0.42 | 1.0 |
| trpD | 6 | 0.096 | 0.59 | 0.66 |
| trpB | 5 | 0.026 | 0.56 | 0.9 |
| gltB | 9 | 0.56 | 0.76 | 0.36 |
| ppc | 4 | 0.0 | 0.54 | 1.0 |
| mdh | 4 | 0.0 | 0.54 | 1.0 |
| pckA | 4 | 0.0 | 0.54 | 1.0 |
| speE | 1 | 0.0 | 0.39 | 0.0 |
| nadD | 1 | 0.0 | 0.39 | 0.0 |
| betB | 2 | 0.0 | 0.5 | 1.0 |
| amiE | 7 | 0.31 | 0.62 | 0.33 |
